# Internet-based Prescription of Sildenafil: A 2104-Patient Series

**DOI:** 10.2196/jmir.3.1.e2

**Published:** 2001-01-31

**Authors:** Miles J Jones

**Affiliations:** ^1^Consultative & Diagnostic Pathology, IncLee's Summit MOUSA

**Keywords:** Impotence, Medical History Taking, Prescriptions, Drug, Questionnaires, Internet, Commerce, Quality of Health Care, Side Effects, Sildenafil, Physician-Patient Relations

## Abstract

**Background:**

The Internet is becoming increasingly important as a way for patients to acquire medical information and as a means for patient-physician communication. Questions about appropriate use of this new technology have been brought to the fore by the many patients using the Internet to seek sildenafil prescriptions.

**Objective:**

To present the first description of a physician designed and directed Internet-based prescribing system of sildenafil, together with data covering more than 2,100 patient encounters.

**Methods:**

Retrospective analysis of a large case series from a medical practice that prescribes sildenafil based on medical and sexual histories obtained through a physician designed and directed World Wide Web (WWW) site, compared against patients from clinics at a Midwestern inner city medical center. We compared all 2,104 Internet patients seeking sildenafil prescriptions online between June 14, 1998, and March 1, 1999, with all 36 medical center patients obtaining sildenafil prescriptions during the same period.

The outcome measures compared were: completeness of medical record; patient safety as noted by the follow up responses of all patients requesting refills, any comments received by the internet site (webmaster), and patient or physician comments noted in the clinic medical record; satisfaction as noted by the follow up responses of all patients requesting refills, any comments received by the internet site (webmaster), and patient or physician comments noted in the clinic medical record; examinations and laboratory tests.

**Results:**

Fifty-six percent of Internet requests came from 46 states, and 44% from eight foreign countries. Of 2,104 requests, 2,100 were granted. Three hundred ten patients have requested medication refills: all reported erections sufficient for intercourse and 69% said their satisfaction exceeded all expectations; none were at all dissatisfied. Side effect rates were comparable to those in the literature. Comparison of the medical history obtained from Internet patients with that recorded in clinic patients' charts revealed that the former was far more complete. No clinic patient received any examination or laboratory test specific for erectile dysfunction or its causes. There were no reported deaths or serious complications in either group.

**Conclusions:**

Internet-based prescription of sildenafil provides the physician with a complete and very detailed medical and sexual history for 100% of patients without denying any information routinely obtained in a direct patient contact setting. Internet-based practice, which may be expected to require far fewer healthcare resources than traditional settings, rates very high in patient satisfaction among patients requesting a refill; no negative comments were received from all other patients. Overall, these data support the safety and effectiveness of Internet prescribing of selected medications.

## Introduction

Erectile dysfunction is an extremely common condition: according to the Massachusetts Male Aging Study, 52% of surveyed men aged 40 to 70 had some degree of erectile dysfunction, with dysfunction being moderate to complete in approximately half of the 70-year-old men [1]. Similarly, the National Health and Social Life Survey found erectile dysfunction reported by 18% of men aged 50 to 59 (the oldest group surveyed) [2]. Until recently, the condition was often ignored. This reflected both the mistaken perception that nothing practical could be done, and the understandable discomfort of many patients and healthcare providers in openly and frankly discussing sexual matters.

This changed dramatically with the March 1998 approval of sildenafil (Viagra, Pfizer), the first effective oral medication for treatment of erectile dysfunction. Within two weeks, newspapers reported physicians were writing 15,000 to 20,000 prescriptions a day for the medication [3]. Erectile dysfunction had graduated from secret shame to headline news.

Even with erectile dysfunction on the front pages, many men continue to feel uncomfortable discussing their own conditions face-to-face with a health-care provider [4]. At the same time, many patients are turning to the Internet for medical information and advice on a wide variety of conditions, often because they find the relative anonymity of the Internet less intimidating than a traditional office-based consultation [5]. Their Internet usage may take the form of formal physician consultation [6,7]; informal physician-originated information and advice [8,9]; or information, advice, and support from others with similar conditions [10].

This combination of circumstances suggests the Internet as a natural resource for addressing the needs of men with erectile dysfunction who may be reluctant to seek help from their regular physicians. To meet this need, the NET Doctor Group (an association of physicians, pharmacists, and information specialists) established an Internet-based system to provide information on erectile dysfunction and its treatment with sildenafil; to obtain the medical histories of patients with this condition, when appropriate and desired by the patient; and to prescribe sildenafil and, if requested, dispense it through an independent licensed pharmacy. We report on the first 2,104 patients to request sildenafil prescriptions, together with a comparison group of patients who received sildenafil prescriptions in a traditional medical practice (inner city teaching hospital clinic) setting, during the same 10.5 month period.

## Methods

### Setting

The NET Doctor Group is a private company that uses a physician-designed World Wide Web (WWW) site (http://www.net-dr.com) to collect patient information and medical history relevant to prescription of sildenafil. This form is shown in Textbox 1. Physicians associated with but not employed by the NET Doctor Group review the provided medical history, on a fee-for-service basis (the fee is waived if the requested prescription is refused). All physicians are United States educated and trained, hold active license in multiple states, have current DEA registrations, practice independently of the NET Doctor Group, provide full licensure and identification information to dispensing pharmacies, and maintain individual professional liability insurance. The physicians also attempt to make telephone contact with all patients requesting sildenafil prescriptions; such contact is required when the submitted information appears contradictory or otherwise inadequate to support a decision. Requests are typically approved or refused within 24 hours of submission.

U.S. patients who are approved for sildenafil prescription have a choice of receiving the medication directly from the NET Doctor Group's independent pharmacy (not owned or operated by the NET Doctor Group), or of having the prescription faxed to their usual pharmacy. Non-U.S. patients receive the medication directly from the NET Doctor Group. All prescriptions are written for 100-mg tablets of sildenafil as follows:

Use 1/2 tab po at least 30 min. before anticipated intercourse (may need to take up to 2 hours before intercourse for maximal effect). If effect inadequate try 1 tab po as above. Warning: Do not take more than 1 tab/day. If unusual pain or symptoms occur consult physician. If chest pain occurs report immediately to nearest ER.

For unusual pain or symptoms, patients are advised to consult a physician. Patients are free to contact any physician, including the NET Doctor Group associated physicians, at any time. The patient insert (see Textbox 2) advises the patient to consult his or her personal physician. For the most severe and life threatening reactions associated with vigorous or infrequent sexual activity, patients are unequivocally advised to report immediately to the nearest ER.

The patient informational insert developed specifically for the NET Doctor Group's patients and included with directly dispensed medication is shown in Textbox 2. The patient information sheet used by the private pharmacy for its non-NET Doctor Group clients is shown in Textbox 3. The typical prescription written for non-NET Doctor Group patients is for 25 mg sildenafil and reads as follows: Take as directed.

**Online Medical Consultation Form**

Please take the necessary time to carefully and truthfully complete all applicable questions on the form. Incomplete forms will not be submitted to our physicians for a consultation, as failure to provide the medical information required to render a professional opinion is prima facie grounds to deny a prescription. If you want to ask us any questions before submitting your form, see our Frequently Asked Questions or email us at inquiries@Net-Dr.com.

It would help us a lot to expedite the consultation process if you provide us your correct email address.

Name:

Address:

City: State: Zip Code:

Country :

Phone: Fax:

Email address:

If my responses indicate that my impotence may be treatable with medication, I understand that Net Doctor physicians have found Viagra to be effective in many cases. If my sexual and medical history suggests that I may benefit from Viagra therapy, please: mail the Viagra tablets immediately.

Please send me a pill cutter. I understand my credit card will be billed.

*Click here for refill information*

**Section A. ( To be completed by All patients )**

date of birth: (mm/dd/yy) height weight lbs.

Sex: male female

Have you been diagnosed with:

atherosclerosis yes no

heart attack yes no

low testosterone yes no

endocrine disorders yes no

diabetes yes no

stroke yes no

prostate cancer yes no

kidney disease/renal failure yes no

hypertension yes no

spinal cord injury yes no

cirrhosis of the liver yes no

thyroid disease yes no

enlarged prostate yes no

liver disease/other yes no

hepatitis yes no

anxiety yes no

Are you on dialysis? yes no

Have you had an organ transplant? yes no

Please list all medications you are now taking:

Please respond to these questions regarding specific contraindicated medications:

Are you taking nitroglycerine? no yes

Are you taking erythromycin? no yes

Are you taking ketoconazole? no yes

Are you taking cimetidine? no yes

Are you taking itraconazole? no yes

Are you taking mibefradil? no yes

List of Contraindicated Medications

Please list all known allergies:

Are you being treated for other medical conditions at this time? no yes (please specify)

What is your past surgical history?

Do any diseases/disorders run in your family? no yes (please specify)

Describe your alcoholic beverage consumption:

Have you ever been treated for substance abuse? no yes

If you answered "yes", please describe:

Do you smoke? no yes packs per day

Do you consider anything else in your medical history to be relevant? no yes (please specify)

**Section B (To be completed by patients seeking Viagra)**

If you are a woman, describe why you are interested in taking Viagra: (you must have a qualifying medical reason, Viagra is not a recreational drug). Women may obviously leave blank all impotence-related questions which follow, please answer all other questions.

How would you describe your sex life?

Impotence occurred: Suddenly Gradually

Impotence involves: inability to maintain an erection inability to get an erection

Which best describes your impotence:

penis not at all firm

penis not self-supporting but slightly firm

hardness sufficient for sex but firmness is decreased

Are there ever times when you are not impotent? no yes (please specify)

Have you consulted any other physicians about erectile dysfunction? no yes

If you answered 'yes', please indicate the type of treatment you received:

medical surgical injectable psychological

If you have been treated, were you satisfied with the results? Please explain:

Are you interested in trying Viagra for your erectile dysfunction? yes no

Do you suffer from depression? no yes (please specify frequency and severity)

Do you have any of the following conditions:

Multiple Myeloma no yes

Sickle cell anemia no yes

Curvature of the penis (Peyronie's disease) no yes

Leukemia no yes

Bleeding disorders no yes

Active peptic ulcer no yes

Retinitis pigmentosa no yes

Viagra should be taken with caution and only under the direct supervision of your local physician if you suffer from heart complications or have a family history of heart problems. The following questions are designed to determine that risk:

Do you have a pacemaker? no yes

Have you been diagnosed with angina pectoris? no yes

Do you or have you suffered from chest pains? no yes

Do you suffer from shortness of breath? no yes

Do you exercise regularly? no yes

If you answered "yes", please describe the nature/duration of your exercise:

Are your natural mother and father alive? no yes

If you answered "no", please provide any health reasons that contributed to the death of your mother/father:

How long has it been since you were last sexually active?

I have read and understand the side effects of Viagra true false

By submitting this form, I certify that:

I understand that my credit card will be billed for a physician's consultation if I am approved for Viagra therapy. (If you are not approved, there is no charge.) true false

I have read and agree to the Net Doctor's waiver of liability true false

I am permitted to receive Viagra in the state/region/country indicated as the shipping address: true false

Note to US patients: In many cases, a Net Doctor physician shall prefer to speak on the telephone briefly with patients prior to reaching a final decision. Please provide a telephone number (if different than above) where you may be reached and a convenient time in the 24 hours following the submission of your form. If you are not available at the time that the physician phones you, no message will be left, and if someone other than the patient answers the phone no details about the nature of the call or the caller will be disclosed. The physician will try to phone you on the following day at the same time, and if you still cannot be reached, we will contact you via e-mail.

Phone number (if different than above): (US patients only)

Convenient time to call:

Where did you find us?

Net Doctor Home || FAQs || Privacy & Security || Contact Us

Copyright 1998 The Net-Dr. Group All rights reserved.

Revised: April 24, 1999.

**Internet Viagra Patient Information Sheet**

**Viagra 100mg Tablets**

Take 1/2 tablet by mouth at least 30 min. before anticipated intercourse(may need to take up to 2 hours before intercourse for maximal effect). If effect inadequate try 1 tablet by mouth as above. Warning: Do not take more than 1 tab/day. If unusual pain or symptoms occur consult physician. If chest pain occurs report immediately to nearest emergency room.

IMPORTANT!!! Please read all of the following information concerning the medication, including the list of medications that should not be taken with Viagra.

*What is Viagra used for?*

Viagra is used to treat impotence in men. Viagra increases the body's ability to achieve and maintain an erection during sexual stimulation. Viagra does not protect you from getting sexually transmitted diseases, including HIV.

*Who should not take Viagra?*

Men who are currently using medicines that contain nitrates (see complete list below) should not use Viagra because taken together they can lower the blood pressure too much. Other medications may also interact with Viagra. Please review the list below. Viagra should not be used by women or children.

*General Precautions with Viagra:*

Men who have medical conditions that may cause a sustained erection such as sickle cell anemia, leukemia or multiple myeloma or who have an abnormally shaped penis may not be able to take Viagra. There are several medications that are known to interact with Viagra, so be sure to tell your doctor and pharmacist about all medications you are taking including those you can get without a prescription. In addition, please review the list below of medications that should not be taken with Viagra.

*How should I take Viagra?*

Your physician has prescribed Viagra as one tablet about 1 hour before sexual activity. However, Viagra may be taken anywhere from 30 minutes to 4 hours before sexual activity. Do not take more than one tablet in a 24 hour period.

*What are some possible side effects of Viagra?*

Viagra is generally well tolerated. If any side effects are experienced, they are usually mild and temporary. The following is a listing of the most common side effects(This list is NOT a complete list of side effects reported with Viagra. Your personnal pharmacist or physician can discuss with you a more complete list of side effects.): Headache Flushing Upset stomach Diarrhea Stuffy nose Urinary tract infection Visual changes such as mild and temporary changes in blue/green colors or increased sensitivity to light.

*IMPORTANT!!! Medications to Avoid when taking Viagra:*

Amyl Nitrate Cardilate (Erythrityl tetranitrate)

Cartrax (Pentaerythritol tetranitrate)

Dilatrate & Dilatrate SR (Isosorbide dinitrate)

Diflucan (Fluconazole)

Duotrate (Pentaerythritol tetranitrate)

Erythromycin Imdur (Isosorbide mononitrate)

Ismo (Isosorbide mononitrate)

Iso-Bid (Isosorbide dinitrate)

Iso-D (Isosorbide dinitrate)

Isordil (Isosorbide dinitrate)

Isotrate (Isosorbide dinitrate)

Miltrate & Miltrate 10 (Pentaerythritol tetranitrate)

Minitran Transdermal System (Nitroglycerin Patches)

Monoket (Isosorbide monnitrate)

Nitro-Bid (Nitroglycerin)

Nitro-Dur (Nitroglycerin)

Nitro-Time (Nitroglycerin)

Nitrong (Nitroglycerin)

Nitroguard (Nitroglycerin)

Nitrol Ointment (Nitroglycerin)

Nitrocine (Nitroglycerin)

Nitroglyn (Nitroglycerin)

Nitrolingual Spray (Nitroglycerin)

Nitropress (Sodium nitroprusside)

Nitrostat (Nitroglycerin)

Nizoral (Ketoconazole)

Onset-5 (Isosorbide dinitrate)

Papavatral (pentaerythritol tetranitrate)

Pennate (pentaerythritol tetranitrate)

Penta Cap#1 (pentaerythritol tetranitrate)

Pentrate (pentaerythritol tetranitrate)

Pentritol (pentaerythritol tetranitrate)

Peritrate (pentaerythritol tetranitrate)

Sorbide-10 (isosorbide dinitrate)

Sorbitrate & Sorbitrate SR (isosorbide dinitrate)

Tagamet (Cimetidine)

Tetrate-30 (Pentaerythritol tetranitrate)

Transderm Nitro (Nitroglycerin)

Transerm-NTG (Nitroglycerin)

Tridil (Nitroglycerin)

**Walk-in Patient Sildenafil Information Sheet**

USES: This medication is used to treat male sexual function problems (erection problems).

HOW TO USE THIS MEDICATION: This drug is taken by mouth as needed between four hours and one-half hour before sexual activity (about one hour before is most effective). Take only as directed, usually once daily as needed. Sildenafil works along with sexual stimulation to help achieve an erection.

SIDE EFFECTS: Headache, flushing, stomach upset, nasal stuffiness, diarrhea and dizziness might occur. If these effects persist or worsen, notify your doctor promptly. Unlikely but report promptly any pain or other urination problems, vision problems or skin rash. Very unlikely but report promptly any chest pain, fainting or foot/ankle swelling.

PRECAUTIONS: Before using this drug, tell your doctor your medical history, including any allergies (especially drug allergies), any penis conditions such as fibrosis/scarring, history of painful/prolonged erection (priapism), sickle cell anemia, blood system cancers (such as leukemia or myeloma), or Peyronie's disease, eye problems (retinitis pigmentosa), kidney or liver disease, bleeding disorders or active stomach ulcers. Limit alcohol intake, as it may aggravate side effects or this drug. Since this drug may cause dizziness, caution is advised when performing tasks requiring alertness (e.g. driving). To avoid dizziness and lightheadedness when rising from a seated or lying position, get up slowly. This drug is not to be used in women or children. The elderly may be more sensitive to the side effects of this drug, therefore caution is advised in this group.

NOTES: Do not share this medication with others, since they may have a problem that is not effectively treated by this drug. Use of this drug does not protect against sexually transmitted disease.

MISSED DOSE: Not applicable.

STORAGE: Store at room temperature between 59 and 86 degrees F (15 - 30 degrees C) away from light and moisture.

### Patients

This study includes all patients of the NET Doctor Group who requested prescriptions for sildenafil during the period from the opening of the Web site on June 14, 1998, to March 1, 1999. All patients acknowledged a waiver of liability and agreed to specific terms of comprehension and truthfulness before submitting their request for physician consultation. The wavier applies to the NET Doctor Group, and not the independent physicians associated with the NET Doctor Group, as they are not employees of the NET Doctor Group. Each physician may rely on the patient's understanding of sildenafil and its potential complications, and the veracity of the patient's answers to the data collection device, as implied by the wavier.

We informally compare these Internet patients to patients receiving sildenafil prescriptions at clinics of an inner city teaching hospital during the same period. We reviewed charts of these patients, most of whom had been seen on several occasions prior to prescription of sildenafil, for a period of up to one year prior to the prescribing visit and for all subsequent visits. This report covers information obtained from the medical records of all patients who received sildenafil prescriptions as a result of in-person clinic consultations. It should be noted that the clinics are part of an inner city teaching hospital that is a publicly owned and operated facility. Thus, most clinic patients are members of a lower socioeconomic group. Most patients of the hospital do not have the financial means to afford treatment for erectile dysfunction. The small number of patients (36) in the office-based comparison group is a reflection of the group's socioeconomic status.

## Results

Characteristics of patients receiving sildenafil prescriptions are reported in Table 1. During the period covered by this report, 2,104 patients requested sildenafil prescriptions at the NET-Doctor Web site. These patients were somewhat younger than typical for men with erectile dysfunction (mean age 49.4 + 5.3 years, median age 45.9 years), possibly reflecting a lower rate of computer usage among the elderly. Diagnoses of hypertension were reported by 18%, of diabetes by 13%, and of atherosclerosis by 7%. Ten patients had been treated for prostate cancer and one had experienced a spinal cord injury. Approximately 33% of the patients said they smoked; very few reported more than social drinking, while an unusually high 86% reported exercising regularly.

About 95% of the patients said that their impotence had occurred gradually, and 97% that it involved an inability to maintain, rather than to achieve, an erection. Most said their penises were slightly firm but not self-supporting, although a significant minority reported hardness sufficient for sex despite decreased firmness. Eighty seven percent indicated that there were times they were not impotent.

Significantly, almost 66% of the patients reported previously seeking treatment from other physicians for their erectile dysfunction. At least 75% of these men received only psychological counseling or reassurance, and almost none were satisfied with their previous treatment.

Of the 2,104 requests for sildenafil, 2,100 were granted. The small number of refused requests may appear unusual. It must be remembered that patients requested sildenafil only after reading information on the drug and its contraindications and completing an extensive medical history form. Individuals with contraindications to sildenafil presumably did not complete and submit the form, and therefore do not appear in the database of patients requesting sildenafil.

Three of the requests that were refused came from the same address, provided the same demographic information, and requested the maximum number of tablets (30) for a single prescription. Attempts to reach the requester or requesters by telephone were unavailing. The other patient whose request was refused reported having been diagnosed with stroke, hypertension, and angina, yet denied chest pain. Attempts to reach him by telephone were likewise unavailing. Attempts were made to contact all patients by telephone. Less than 10% of all patients had conversations with the consulting physician. Less than one dozen questions required a physician's response; all questions were answered by email within 24 hours. The questioning patients submitted no follow-up questions.

**Table 1:** Characteristics of Internet Patients Receiving Sildenafil Prescriptions

**Category**	Number	Percent	Clinic Chart Documentation
All Patients	2100	100	X
Male	2097	99.90	
Female	3	0.10	
Place of Residence:			
U. S. (46 states)	1167	76	X
Non-U.S. (8 countries)	933	24.0	
Age (mean +/- SD)	49.4 +/- 5.3		X
Height (mean +/- SD)	177.8 +/- 11.2		X
Weight (mean +/- SD)	82.4 +/- 15.6		X
Diagnosed with:			
Atherosclerosis	157	7.50	
Endocrine disorders	51	2.40	
Diabetes	268	12.80	
Stroke	1	0.04	X
Prostate cancer	18	0.50	
Enlarged prostate	152	7.20	
Kidney disease/renal failure	8	0.40	
On renal dialysis	1	0.04	
Hypertension	384	18.30	X
Spinal cord injury	1	0.04	
Cirrhosis of liver	2	0.10	
Hepatitis	1	0.04	
Thyroid disease	1	0.04	
Anxiety	10	0.50	
Depression	1	0.04	
Currently taking:			
Nitroglycerine	0		
Erythromycin	0		
Ketoconazole	0		
Itraconazole	0		
Cimetidine	0		
Mibefradil	0		
Being treated for other medical conditions	46	2.20	
Allergies (various)	61	2.90	
Past surgical history (various)	179	8.50	
Regular exercise	1806	86.00	
Both mother and father alive	884	42.10	
Alcoholic beverage consumption:			
Never	968	45.70	
Rarely	352	16.80	
Social	652	31.00	
Moderate	124	5.90	
Heavy	12	.060	
Smoking reported	673	32.10	
Packs/Day	1.5 +/-0.7		
Sex life described as:			
Good	149	7.10	
Poor	1951	92.40	
Time since last sexual activity	37 +/-9.4 days		
Impotence occurred:			
Suddenly	117	5.60	
Gradually	1983	94.40	
Ever not impotent	1827	87.00	
Impotence involves inability to:			
Maintain an erection	2037	97.00	
Get an erection	63	3.00	
Impotence described as:			
Penis not at all firm	63	3.00	
Penis slightly firm, not self supporting	1267	60.30	
Firmness decreased but sufficient for sex	770	36.70	
Previous treatment for erectile dysfunction:			
Medical	26	1.20	
Surgical	25	1.20	
Injectable	34	1.60	
Psychological	1009	48.00	
Not specified	286	13.60	
Satisfied with previous treatment	15	0.70	
Have following conditions:			
Multiple myeloma	0		
Sickle cell anemia	0		
Peyronie's disease	0		
Leukemia	0		
Bleeding disorders	0		
Active peptic ulcer	0		
Retinitis pigmentosa	0		
Pacemaker in use	0		
Angina pectoris	0		
Chest pain (past or present)	0		
Shortness of breath	0		

There were 2,101 male patients and 3 female patients in the group. One of the female patients reported a complete absence of libido, which had been relieved by taking sildenafil. The second had experienced sexual dysfunction since her complete hysterectomy three years previously. The physician managing her case had recommended that she try sildenafil in addition to the estrogen-testosterone combination with which she was currently being treated. The third reported problems of sexual performance. She was prescribed 10 sildenafil tablets.

**Table 2:** Measurements and Conditions Noted in Medical Records of Clinic Patients Receiving Sildenafil Prescriptions

**Category**	Number	Percent	Noted by Net-Dr.com Documentation
All Patients	36	100	X
Male	36	100	X
Female	0	0	X
U.S. Residence	36	100	X
Blood pressure	34	94.40	
Pulse	30	83.30	
Respiration	10	27.80	
Age	36	100	X
Height	6	16.70	X
Weight	6	16.70	X
General examination	20	55.6	
Rectal examination	0	0	
Referral to specialist	0	0	X
Testosterone concentration	0	0	
Diagnosed with:			
Atherosclerosis	0	0	X
Diabetes	0	0	X
Stroke	1	2.70	X
Prostate cancer	0	0	X
Enlarged prostate	0	0	X
Hypertension	4	11.10	X
Currently taking:			
Nitroglycerine	0	0	X
Erythromycin	0	0	X
Ketoconazole	0	0	X
Itraconazole	0	0	X
Cimetidine	0	0	X
Mibefradil	0	0	X
Other medications	16	44.40	X
Being treated for other medical conditions	36	100	X
Allergies (various)	7	19.40	X
Past surgical history (various)	5	13.90	X
Smoking reported	14	38.90	X
Sex life described as:			
Good	0	0	X
Poor	8	22.20	X
Ever not impotent	4	11.10	X

**Table 3:** Information Reported by Patients Requesting Sildenafil Refills

**Category**	Number	Percent
Total seeking refill	310	100
Improvement sufficient for sexual activity	310	100
Improvement sufficient for penetration /intercourse	310	100
Successful sexual performance:		
Always	16	5.20
Almost always	287	92.60
Occasionally	7	2.30
Seldom	0	0
Never	0	0
Overall satisfaction:		
Exceeds all expectations	215	69.40
Meets most expectations	76	24.60
Satisfied	19	6.10
Somewhat dissatisfied	0	0.00
Dissatisfied	0	0.00
Side effects:		
Headache	5	1.60
Diarrhea	0	0.00
Nausea	0	0.00
Dizziness	0	0.00
Nasal congestion	15	4.80
Rash	0	0.00
Urinary tract infection	0	0.00
UTI in partner	2	0.60
Chest pain	0	0.00
Blurred vision	3	1.00
Shortness of breath	0	0.00
Vision - blue/green tinge	9	2.90
Heartburn	9	2.90
Light sensitivity	0	0.00
Skin flushing	17	5.50

Three hundred ten (14.76%) of the 2,100 patients requested refills during the study period and have filled out forms reporting their experiences with the drug (see Table 3). The number of patients granted refills at their pharmacies is unrecorded. All 310 reported improvement sufficient for them to resume sexual activity and to achieve penetration. Two hundred eighty seven reported that they almost always enjoyed successful sexual performance, with 16 claiming that their sexual performance was always successful and 7 stating that successful performance occurred occasionally. Two hundred fifteen said that their overall satisfaction exceeded all expectations, 76 that it met most expectations, and 19 that they were satisfied; none said that they were dissatisfied or somewhat dissatisfied. Reports of side effects listed in Table 3 were comparable to those from sildenafil clinical trials [11]. Since data could be obtained only from patients requesting refills, however, the sample may not be fully representative. No patient has complained directly to the NET Doctor Group via its web site. All prescriptions list the name, address, and telephone number of the dispensing pharmacy. The name of the prescribing physician is included on each prescription and the dispensing pharmacy has the physician's DEA number, state license, office address, and telephone numbers. No pharmacy has contacted the NET Doctor Group or its associated physicians concerning patient complaints.

During this same period, 36 patients obtained sildenafil prescriptions from the hospital clinics. The type of medical information contained in the office-based group's charts is summarized in Table 2. These patients' medical records generally showed that blood pressure and pulse rate had been recorded at least once during the previous six months (including the index visit). Twenty of the 36 patients had received a general physical examination during that period, but only 6 had had their height and weight recorded and there was no record that any had received a rectal examination.

Eight clinic patients were recorded as stating that their sex lives were poor and 4 as saying that there were times they were not impotent; these items were not recorded on any of the other reviewed charts. No chart contained any statement as to the quality of the patient's erection or whether the onset of erectile dysfunction was gradual or sudden. Likewise, no chart recorded either a blood lipid profile or any laboratory test relevant to diabetes. The only medical conditions of note recorded in these patients' charts were 4 instances of hypertension and 1 cerebrovascular accident. Just 16 of the 36 charts included a complete list of medications being taken by the patient. Since the primary contraindications to sildenafil use are certain concurrent medications, this is a significant omission. No deaths or serious complications among patients using sildenafil have been reported to the NET Doctor Group, its associated private physicians or independent pharmacies by any patient; patient family member; attorneys representing the patient or their estate; or any local, state, or federal governmental agency. The medical records of the clinic patients had no indication of any adverse effects or death related to sildenafil usage. Lastly, FDA and Pfizer surveillance systems have not reported any deaths directly attributed to sildenafil. The possibility of unreported deaths or complications cannot be completely ruled out.

## Discussion

The explosive popularity of sildenafil, and the demonstrated desire of many patients to obtain this medication without a face-to-face discussion of what they regard as intimate personal matters, has brought to the fore the simmering question of Internet-based medical advice and consultation [12]. Concern has been expressed by the Food and Drug Administration, members of the American Medical Association's Council on Ethical and Judicial Affairs, and the vice president of the Federation of State Medical Boards [13].

Yet sildenafil is only a small part of the changes currently in progress. At least two groups are offering fee-for-service Internet-based medical consultations on a wide variety of conditions [6,7] and many other physicians find themselves responding to on-line requests for medical information and advice even when that is not their officially stated policy [9]. Indeed, medical information on the Internet is proliferating so rapidly that it has been the subject of an official report from a panel convened by the U.S. Department of Health and Human Services [14]. While this panel did not specifically address one-to-one communication between physicians and patients, it did note many of the benefits, such as greater willingness to engage in frank discussions about health status, behavioral risks, and fears and uncertainties.

### Advantages and problems

Advantages of Internet-based patient-physician communication when real-time contact is not required include convenience for the patient and savings in both time and office resources for the physician. Perhaps most importantly, the computer interface greatly facilitates both obtaining and recording a complete medical history. This conclusion is supported by our comparison of medical history data from the Internet consultation form with that from the charts of clinic patients. Due to the limited number of patients and the limited clinical setting, our findings indicate the need for funding further study and comparisons of physician directed Internet prescribing versus traditional prescribing practice.

One objection that has been raised to Internet-based consultation and prescribing is that, as with the NET Doctor Group, there is generally no mechanism for providing a consultation report to the patient's primary care physician. Several points need to be made. One is that, even in the U.S., it cannot be assumed that all patients have a primary care physician. A second is that many patients - almost half of those seeking sildenafil prescriptions - are from outside the U.S.; they and their physicians may have very different expectations regarding consultation reports. Third and most significantly, a considerable fraction of the patients may have sought Internet-based consultation because they did not want their usual physician to know of their condition or of a specific sildenafil order. For example, they may not wish their wife to know of the order and may not fully trust the discretion of a physician who treats both family members. Although this is regrettable, respect for patient autonomy requires that there should be no attempt to contact the patient's primary care physician without the patient's explicit permission.

This latter point is also relevant to the frequently expressed opinion that it is easier to assess a patient's truthfulness in a face-to-face encounter [15,16]. We are dealing here with patients who have specifically chosen to seek a prescription from someone other than their usual physician. In the absence of an established relationship, mere physical propinquity would do little to assure a complete and truthful medical and sexual history. Indeed, the relative anonymity of the Internet may, in our opinion, well increase patient truthfulness and openness. Another objection sometimes raised is that Internet-based practices, including the one described here, may not specifically list the credentials of the associated physicians [15,17]. However, while physicians reviewing the site might find such information reassuring, its usefulness to patients appears remote. With rare exceptions, very few patients either understand or utilize the data on physician credentials that are available to them. Rather, they typically base their initial choice of physician on friends' recommendations and on convenience. Those factors remain valid in the context of Internet-based prescribing.

### Can erectile dysfunction be managed online?

Clearly, not every medical condition is appropriately managed by Internet encounters alone. Erectile dysfunction may be particularly prominent among the appropriate conditions. Although objective means exist for establishing the existence of erectile dysfunction and for distinguishing between organic and psychogenic causes [18], these tests are cumbersome and often omitted even from specialists' most comprehensive recommendations. Indeed, there appears to be little in the way of consensus as to what, if anything, beyond the medical history might be appropriate in the diagnostic work-up of erectile dysfunction [19]. Recommendations that the physical examination focus on signs of vascular [20] and neurologic [21] disease, together with palpation of the penis for Peyronie's disease [22] and tests for atrophy are common [23]. Except for detection of Peyronie's disease, which can usually be elicited by a thorough medical history, these observations are directed primarily toward determining a cause for the dysfunction. Prior to advent of sildenafil, the etiology of erectile dysfunction rarely affected the choice of treatment [24]. Consequently, Hakim and Goldstein limit their recommended physical examination to abnormal penile curvature and palpable corporal fibrosis [25].

Vinik has noted that erectile dysfunction is often the presenting symptom of diabetes and is also a marker for development of generalized vascular disease and for myocardial infarction [26]. Godschalk et al [23] recommend inclusion of a hemoglobin A1c and a lipid profile in all work-ups for erectile dysfunction. Similarly, Mobley and Baum recommend assessment of sacral root function by means of a rectal examination that includes evaluation of the bulbocavernosus reflex and of sphincter tone [27]. All three tests are absent from the recommended diagnostic work-ups of other experts. Significantly, we found no mention of them in the charts of any of the teaching hospital clinic patients who received a sildenafil prescription.

One can conclude that there is an almost total absence of expert consensus as to the essential components of an erectile dysfunction work-up. The only area of agreement is the importance of a complete medical and sexual history. Our observations of practice in a Midwestern inner city teaching hospital clinic suggest that physicians in this setting rely primarily on the history in deciding whether a sildenafil prescription is appropriate. Yet we also find that the medical and sexual history they obtain is less complete than that obtained by the NET Doctor Group.

Once the physician has concluded that a sildenafil prescription is appropriate, the next step is to instruct the patient in the medication's proper and safe use. Although it is extremely difficult to assess how well different physicians communicate such instructions to their patients, many observations suggest that physician-patient communication is often less than optimal [28]. It would presumably follow that many patients do not understand the instructions they receive.

When oral information is poorly expressed or poorly understood, the patient information sheet becomes critical. As a comparison of Textbox 2 and Textbox 3 shows, the information sheet provided by the NET Doctor Group to its Internet patients is far more thorough and complete, and perhaps more comprehensible as well. Further, it is not the standard for pharmacists to provide a specific patient instruction sheet when dispensing sildenafil. Greater thoroughness may be particularly important given the intuitively plausible assumption that Internet users as a group are likely to grasp information more easily when it is presented in written rather than oral form.

### Are current ethical codes and legislation too restrictive?

The American Medical Association has noted that:

Telecommunications advisory services, by way of phone, fax, or computer, can be a helpful source of medical information for the public. Often people are not sure where to turn for information of a general medical nature or do not have easy access to other sources of information. Individuals may also be embarrassed about directly bringing up certain questions with their physicians [29].

The statement goes on to say: "Under no circumstances should medications be prescribed." This dictum, which is currently being revised [13], appears unduly restrictive. It may derive from the belief that, without physical examination and laboratory tests, the etiologic basis of a patient's complaint cannot be identified. The point overlooked by this assumption is that sildenafil is only one of many medications intended, not to cure an etiology, but to relieve a symptom; symptoms are normally diagnosed solely on the basis of patient histories.

The ease with which the appropriateness of certain medications may be assessed underlies the prescriptive authority sometimes granted pharmacists, who are highly expert in medications and their uses but have little or no diagnostic training. As of 1996, 16 states plus the Indian Health Service and the Department of Veterans Affairs allowed pharmacists to initiate or modify drug therapy under certain conditions; similar legislation was pending in 15 additional states [30]. In almost all instances, this authority was gained with the acquiescence of organized physician groups [31].

Support for pharmacists initiating drug therapy is greatest when the condition being treated is diagnostically obvious. A 1993 survey of New York State internists and family practitioners found that 64% of the physicians questioned supported pharmacists providing a butoconazole vaginal cream for candidiasis and 61% supported pharmacists providing a steroid-containing rectal suppository for hemorrhoid sufferers [32]. Washington State allows certain pharmacists to directly dispense emergency contraceptives [33]. And although Florida pharmacists are authorized to prescribe approximately 30 types of medication, 82% of their prescriptions fall into just three categories: topical pediculicides (lindane shampoos), oral analgesics, and otic analgesics [34].

Neither physicians nor the Food and Drug Administration, which in recent years has approved the transfer of large numbers of formerly prescription-only drugs to nonprescription status [35], believe that every prescription drug calls for elaborate physician physical examination and history. The clinic records from the inner city teaching hospital we examined indicate that some believe sildenafil may belong in this category. It might well be argued that the only reason sildenafil requires a prescription is the need for monitoring of contradictions and potential drug interactions, plus the mistaken ideas many patients hold about its indications and proper usage. This study provides evidence that both monitoring and the provision of patient information can be performed via the Internet at least as well as, and perhaps better than, through a traditional face-to-face physician-patient interview.

### Cross-border issues

Concerns have been expressed about the potential availability to patients of drugs not yet approved in their countries of residence [12]. That this is a very real possibility is shown by the experience of the NET Doctor Group: 44.4% of the sildenafil prescriptions were issued to patients in 8 different non-U.S. countries. The concern is not truly limited to Internet-based prescribing: many Canadians crossed the border to obtain sildenafil from U.S. pharmacies prior to the medication's availability in their own country [36].

A large number of countries allow patients to import small amounts of nationally unapproved medications for their personal use. The question is whether individuals' increased ability to obtain nationally unapproved drugs without physically traveling outside their country of residence calls for changes in the law.

### Conclusions

Our results support Internet-based prescription (IBP) of sildenafil utilizing a physician designed and controlled information and decision system. The Internet-based prescribing physician consistently has more, not less, clinically relevant and useful information than was typically obtained and utilized in a specific hospital clinic setting. The data suggest that contrary too conventional thought, there is no evidence of compromise to patient safety. This statement is made with the important limitation that our study utilized only passive means to document patient adverse reactions or complaints. Our web site is available for comments 24 hours a day every day of the year. No negative feedback from patients using sildenafil was noted. Established monitoring systems operated by the FDA, Pfizer, local and state governments are actively gathering data. As of the current date we have served over 5100 patients and have received only one complaint from a patient (the patient had a history of asthma and was distressed that he was granted a prescription, he had been advised by a physician "acquaintance" it was unsafe for him to use sildenafil). While our patients represent a minute proportion of all patients using sildenafil, we expect the aforementioned external event tracking systems would detect any significant variation in the expected outcomes of our patients. Based on the significant lack of spontaneous and voluntarily recorded complaints and the overwhelmingly positive comments of patients seeking refills, patients appear to be satisfied with our approach. IBP is associated with extremely low demands on health care resources and maximum responsiveness to patient needs. Health care standards and governmental regulatory efforts to date have not been based on objective or experimental evidence. They have significantly lagged behind the capabilities and implementation of Internet prescribing systems. We hope that data from this first large, objective scientific study can serve as a starting point for development of fact based, meaningful standards and regulations. We encourage further and broader evaluation of physician-designed and controlled Internet prescribing systems.
